# Increases in Daily Defined Doses of Incident Benzodiazepine Prescriptions in the Netherlands During the Second and Third COVID-19 Lockdowns

**DOI:** 10.1192/bjo.2024.267

**Published:** 2024-08-01

**Authors:** Damian Visser, Daphne Everaerd, Indira Tendolkar, Janneke Zinkstok, Arnt Schellekens

**Affiliations:** Radboudumc, Nijmegen, the Netherlands

## Abstract

**Aims:**

The aim of this study is to investigate incident and total benzodiazepine prescribing in the Netherlands during the COVID-19 pandemic, including the impact of lockdown periods.

**Methods:**

A national Dutch pharmacological registry was used, investigating extramural psychiatric drug prescriptions, between March 2020 and March 2022. Data included incident and total prescriptions as well as daily defined doses (DDDs) of benzodiazepines. The data covered 96% out of a total Dutch population of 17.5 million people. This was compared with the previous calendar year as a reference expressed as a monthly risk ratio (RR) and was corrected for population growth. Changes over time will be discussed if the RR was above 1.1 or below 0.9.

**Results:**

A total of 13.4 million prescriptions over a period of three years were included of which 5.8% were incident prescriptions. Three lockdown periods were identified during pandemic.

When analysing the total benzodiazepine prescription group, prescriptions and DDDs remained mostly stable throughout the pandemic. A brief relative increase in prescription DDD amounts was found during the second lockdown (RR: 1.11). When viewing the incident benzodiazepine prescriptions, there was a short period between the first and second lockdown when both prescription numbers and DDDs decreased (RR: 0.86 and RR: 0.83 respectively). The DDDs of incident prescriptions increased sharply during the second and third lockdown period and remained elevated between both, with an average RR of 1.13.

**Conclusion:**

Total monthly benzodiazepine prescriptions and DDDs remained mostly stable during the COVID-19 pandemic in the Netherlands. COVID-19 related lockdowns seem to have mainly influenced incident benzodiazepine DDDs dispensed during the second and third lockdown. Increased incident DDDs, but not prescription numbers, imply that new patients on average received larger benzodiazepine prescriptions. The increase in incident prescription DDDs could be indicative of decreased accessibility to (psychiatric) healthcare. It could also have been driven by an increase of the incidence and/or severity of sleep and anxiety symptoms during the second and third lockdown. A better understanding of exact causes and mechanisms behind these changes is relevant in order to limit the psychiatric repercussions of future (inter)national emergencies.

